# Guilding

**Published:** 1881-02

**Authors:** 


### GUILDING.

The *Scientific American* gives the following as an efficient method of plating small articles of steel and brass with gold, silver, and nickel, without a battery. "Guilding by dipping: Distilled water, 17 pints; pyrophosphate of potassa or soda, 28 ounces; solution of hydrocyanic acid ($\frac{1}{5}$ pure acid), $\frac{1}{3}$ ounce; terchloride of gold, $\frac{2}{3}$ ounce. Put 16 pints of the water in a porcelain or porcelain-lined iron vessel, and gradually stir in the pyrophosphate, heat, filter, and let it cool down. Add the gold chloride dissolved in water, and then the hydrocyanic acid. Heat the bath nearly to the boiling point for use. (Hydrocyanic acid, it must be remembered, is very poisonous, and it must be handled accordingly.) When heated the liquid becomes colorless. If a red or violet is developed, add a few drops more hydrocyanic acid. Clean the articles thoroughly; dip them in a strong aqueous solution of mercurous nitrate, then, for a few seconds, in the hot gold bath, rinse in clear water, dry in warm sawdust, and burnish if desired. Silvering by dipping: To a saturated aqueous solution of bisulphite of soda in pure water add solution of nitrate of silver, with constant stirring, until the precipitate at first formed ceases to redissolve. Use the bath cold in a porcelain enamelled iron vessel. Clean and dip as in the gold bath. We know of no satisfactory method of coating with nickel without a battery."

